# Early Virtual-Reality-Based Home Rehabilitation after Total Hip Arthroplasty: A Randomized Controlled Trial

**DOI:** 10.3390/jcm11071766

**Published:** 2022-03-22

**Authors:** Edoardo Fascio, Jacopo Antonino Vitale, Paolo Sirtori, Giuseppe Peretti, Giuseppe Banfi, Laura Mangiavini

**Affiliations:** 1 IRCCS Istituto Ortopedico Galeazzi, 20161 Milan, Italy; edoardo.fascio@gmail.com (E.F.); paosirto@libero.it (P.S.); giuseppe.peretti@unimi.it (G.P.); banfi.giuseppe@fondazionesanraffaele.it (G.B.); laura.mangiavini@unimi.it (L.M.); 2 Department of Biomedical Sciences for Health, University of Milan, 20133 Milan, Italy; 3 Vita-Salute San Raffaele University, 20132 Milan, Italy

**Keywords:** rehabilitation, virtual reality, hip, arthroplasty, exercise, home-based

## Abstract

The benefits of early virtual-reality-based home rehabilitation following total hip arthroplasty (THA) have not yet been assessed. The aim of this randomized controlled study was to compare the efficacy of early rehabilitation via the Virtual Reality Rehabilitation System (VRRS) versus traditional rehabilitation in improving functional outcomes after THA. Subjects were randomized either to an experimental (VRRS; *n* = 21) or a control group (control; *n* = 22). All participants were invited to perform a daily home exercise program for rehabilitation after THA with different administration methods—namely, an illustrated booklet for the control group and a tablet with wearable sensors for the VRRS group. The primary outcome was the hip disability (HOOS JR). Secondary outcomes were the level of independence and the degree of global perceived effect of the rehabilitation program (GPE). Outcomes were measured before surgery (T0) and at the 4th (T1), 7th (T2), and 15th (T3) day after surgery. Mixed-model ANOVA showed no significant group effect but a significant effect of time for all variables (*p* < 0.001); no differences were observed in HOOS JR between VRRS and the control at T0, T1, T2, or T3. Further, no differences in the level of independence were found between VRRS and the control, whereas the GPE was higher at T3 in VRSS compared to the control (4.76 ± 0.43 vs. 3.96 ± 0.65; *p* < 0.001). Virtual-reality-based home rehabilitation resulted in similar improvements in functional outcomes with a better GPE compared to the traditional rehabilitation program following THA. The application of new technologies could offer novel possibilities for service delivery in rehabilitation.

## 1. Introduction

Hip osteoarthritis is a common musculoskeletal degenerative disorder in adulthood resulting in higher functional disability and lower quality of life, and can also ultimately lead to higher healthcare costs [1]. The incidence of degenerative diseases increases with age; nonetheless, the elderly now have high standards in relation to their independence, sociality, and quality of life [2]. In this context, the ability to walk is crucial for many daily activities, and an increasing number of older individuals undergo orthopedic surgery to restore mobility [3]. For this reason, total hip arthroplasty (THA) has become one of the most common procedures in orthopedic surgery [4,5], with an increase in the use of this surgical procedure of approximately 55% over the last decade [6].

The COVID-19 pandemic has revealed the disfunction of the systems in different economic sectors, with many having to cope with sudden digitization. The Italian National Health System has seized the opportunity to modernize itself, activating many e-health services and encouraging the use of telemedicine. In fact, in December 2020, after a long normative preparation, the conference between the Italian state and its regions approved the national guidelines on telemedicine [7]. According to the Italian National Plan of Recovery and Resilience (PNRR) [8] and NextGenerationEU, telemedicine represents an extraordinary tool to strengthen the health system coverage at the local level to ensure a better experience for patients and improve the efficiency of regional health services. Over the years, parallel to the institutions, many organizations from different countries have tested various types of telemedicine services, producing a base of scientific literature on the efficiency and effectiveness of the implementation of e-health services, such as telerehabilitation [9]. In the field of orthopedic rehabilitation, various systematic reviews [10] and meta-analyses [11,12,13,14] have shown similar clinical outcomes after face-to-face rehabilitation as compared to telerehabilitation. In Italy and worldwide, the number of joint replacement surgeries is increasing sharply; of these, hip arthroplasty is the most widely practiced, and it has been shown to be effective in ameliorating the health-related quality of life of subjects diagnosed with end-stage osteoarthritis [15,16]. Rehabilitation plays an important role in the improvement of patient quality of life; nevertheless, the need to analyze the efficacy and effectiveness of the rehabilitation protocols, which must be specific, prompt, and reproducible, is increasingly being felt [17]. To date, the benefits of early virtual-reality-based home rehabilitation following THA have not yet been assessed. It is our aim to advance the knowledge of the provision of health services by means of Internet communications technologies (i.e., telerehabilitation). It is possible that implementing a telerehabilitation system could bring many advantages, such as improvements in patient compliance with prescriptions, better patient monitoring, and cost savings. Therefore, the aim of this study is to compare the efficacy of early rehabilitation via the Virtual Reality Rehabilitation System (VRRS) versus traditional rehabilitation in improving functional outcomes after primary THA.

## 2. Materials and Methods

### 2.1. Study Design

An open-label, parallel, two-group, randomized controlled trial (RCT) was conducted between 1 March 2019 and 16 February 2021 at Galeazzi Orthopedic Institute. The study was approved by the Ethics Committee of Vita-Salute San Raffaele University of Milan (147/int/2018), and all study procedures were performed in accordance with the Declaration of Helsinki. All subjects received clear explanations of the purpose, methods, potential risks, and benefits of the study, and before the beginning of the experimental procedures, written informed consent was obtained from all participants. The study protocol was previously registered to ClinicalTrials.gov (NCT04221425) and conducted in accordance with the CONSORT guideline.

### 2.2. Sample Size

A difference of 12 points on the Hip Dysfunction and Osteoarthritis Outcome Score for Joint Replacement (HOOS JR) scale between the two groups at the 15th post-operative day was considered clinically significant [18]. Therefore, considering a standard deviation (SD) of 12.45, a 5% type I error, and a 20% type II error, while taking into account a 20% dropout rate from the whole sample, a total sample size of 44 subjects was planned, with 22 subjects in each group.

### 2.3. Participants

**Figure 1:**
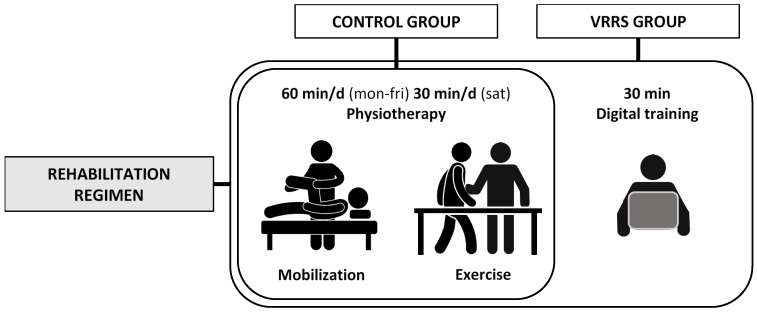
Rehabilitation regimen after THA. During the inpatient post-operative period, patients received 60 min/d (Monday to Friday) and 30 min/d (Saturday) of physiotherapy, consisting of therapeutic exercise and passive mobilization. Only the VRRS group received 30 min of digital training on the use of the VRRS home rehabilitation system in addition to the physiotherapy.

At the pre-admission testing for THA, outpatients aged between 50 and 70 years old with primary or secondary hip osteoarthritis were invited by the principal investigator (GP) to voluntarily participate to the trial. Participants were supposed to have had at least middle school graduation, a home internet connection, and a caregiver. Recruitment proceeded following the outpatients’ order of presentation and their acceptance of the written informed consent until the sample size was reached. A 1:1 ratio randomization list used to allocate each patient either to an experimental (VRRS; virtual reality rehabilitation system group) or to a control group (control) was entrusted to an independent investigator. The exclusion criteria were: congenital or post-traumatic morphological alterations of the hip, neurological or oncological diseases, wearing electronic devices susceptible of electromagnetic fields, epilepsy, being treated with anticoagulant or immunosuppressive therapies, and pregnancy. During a four-night hospitalization period after the surgery, both VRRS and control groups received an identical rehabilitation protocol administered by the same rehabilitation team. Only participants allocated in the experimental group additionally received a half-hour training session for the use of the telerehabilitation device. Figure 1 summarizes the inpatient rehabilitation regimen for both groups.

### 2.4. Virtual Reality Rehabilitation System (VRRS)

**Figure 2:**
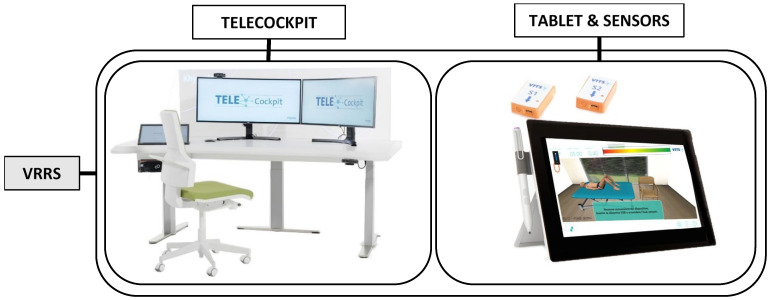
VRRS components. The VRRS telecockpit is the operator workstation used for creating rehabilitation programs, following the progression of the performances, and receiving feedback from the user via live videocalls and therapeutic sessions. The VRRS tablet provides a home virtual rehabilitation program to patients and works in tandem with wearable sensors.

The VRRS (Khymeia Group, Padova, Italy) is a system used for home rehabilitation consisting of an operator workstation and a mobile kit for the user. The operator can manage all portable devices remotely with the VRRS telecockpit, creating a suited rehabilitation program, following the progression of the performances, and receiving feedback from the user via live videocalls and therapeutic sessions. The user is equipped with a tablet (VRRS tablet) that works in combination with wearable sensors for the execution of a virtual exercise program. Quantitative and qualitative data regarding the rehabilitation sessions are registered by the wearable sensors, stored on the tablet, and shared with the operator at the workstation, in compliance with the European privacy policies. Figure 2 shows the VRRS components.

### 2.5. Exercise Program

Participants in both groups were invited to perform a home exercise program daily for the rehabilitation of the lower limbs after THA. The core exercise program was the same for both groups: active mobilization of the operated hip in the sagittal plane avoiding rotation and extension, strengthening of gluteal and tight muscles, and load and balance management. The methods of administration of the exercise program differed between groups, involving an illustrated booklet for the control group and the VRRS tablet for the experimental group. Table 1 details the home rehabilitation regimen used for both groups.

### 2.6. Clinical Outcomes and Assessment

The primary outcome of the study was the hip disability (HOOS JR, 0–100) on the 15th day after surgery. Secondary outcomes were the level of independence (Functional Independence Measure (FIM) 18–126 [19], Modified Barthel Index (BIM, 0–100) [20]) and the degree of perceived effect of the rehabilitation program (global perceived effect (GPE) [21]). The GPE scale asked the participant to rate from 1 to 5 (1: “It worsened the situation”; 2: “It didn’t help me”; 3: “It helped me a little”; 4: “It helped me“; 5: “It helped a lot”) the question “How much did the rehabilitation program help for your recovery?”. The clinical assessments began the day of the pre-admission testing (T0), and they were subsequently performed on discharge from hospital (T1, 4 ± 1 days after surgery) and after a few days (T2, 7 ± 2 days after surgery), ending concurrently with the removal of sutures (T3, 15 ± 2 days after surgery). Assessors were blinded to group allocation. The GPE was measured only at T3, whereas FIM and Barthel were not measured at T2.

### 2.7. Statistical Analysis

Data analysis was performed using SPSS software (IBM^®^ SPSS^®^ Statistics 20) and interpreted at the two-tailed significance level of <0.05. Demographic characteristics are reported as absolute and relative frequencies for categorical variables and as means with SDs for continuous variables. The normality of outcome measures was assessed at each time point and for the two study groups separately with the Shapiro–Wilk test; in accordance, parametric (*t*-test) or non-parametric (Mann–Whitney U test) methods were applied to compare groups at baseline. The chi-square test was used to assess significant associations between categorical variables. Analysis of variance (ANOVA) was used to test whether there was an interaction between time (within-group factor) and type of intervention (between-group factor); furthermore, Tukey’s multiple comparison was used. Both per-protocol and intention-to-treat analyses (using multiple imputations to impute values for the missing data) [22] were performed.

## 3. Results

### 3.1. Participants

**Figure 3:**
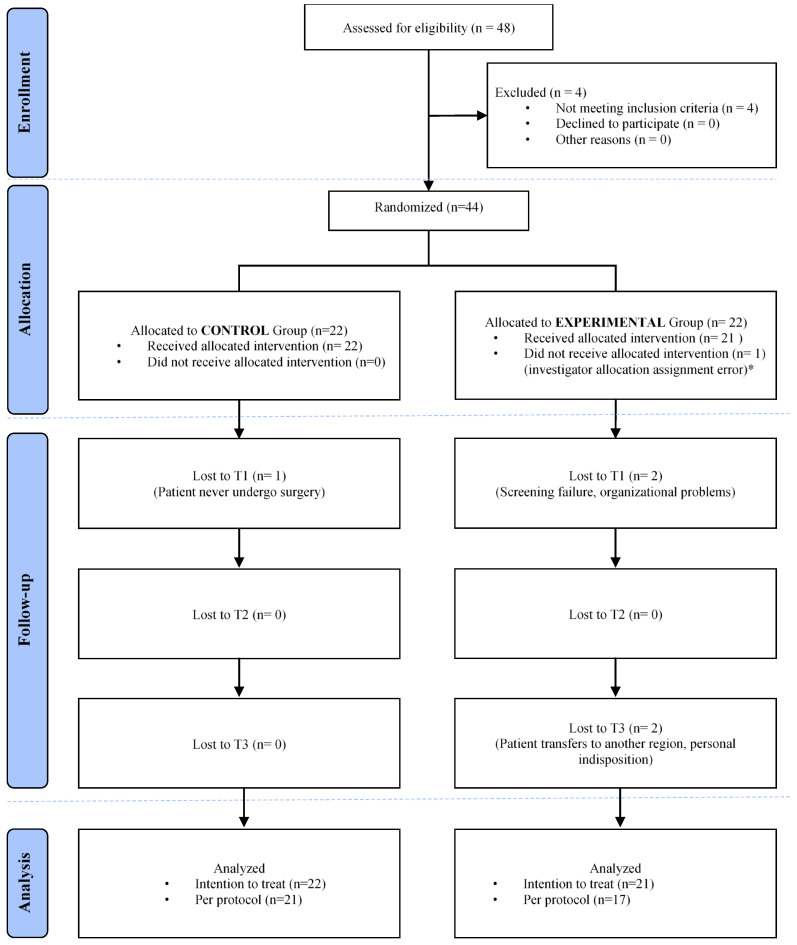
Study flow chart (CONSORT, 2010). * Following the allocation to the experimental group, one participant erroneously received the intervention assigned to the control group.

Overall, 93.4% of the variables were collected from 44 participants, whereas 6.6% of the missing data were imputed by means of the multiple-imputation analysis method. The baseline characteristics were similar for both groups (Table 2). Figure 3 shows the study flow chart.

### 3.2. Outcomes

**Figure 4:**
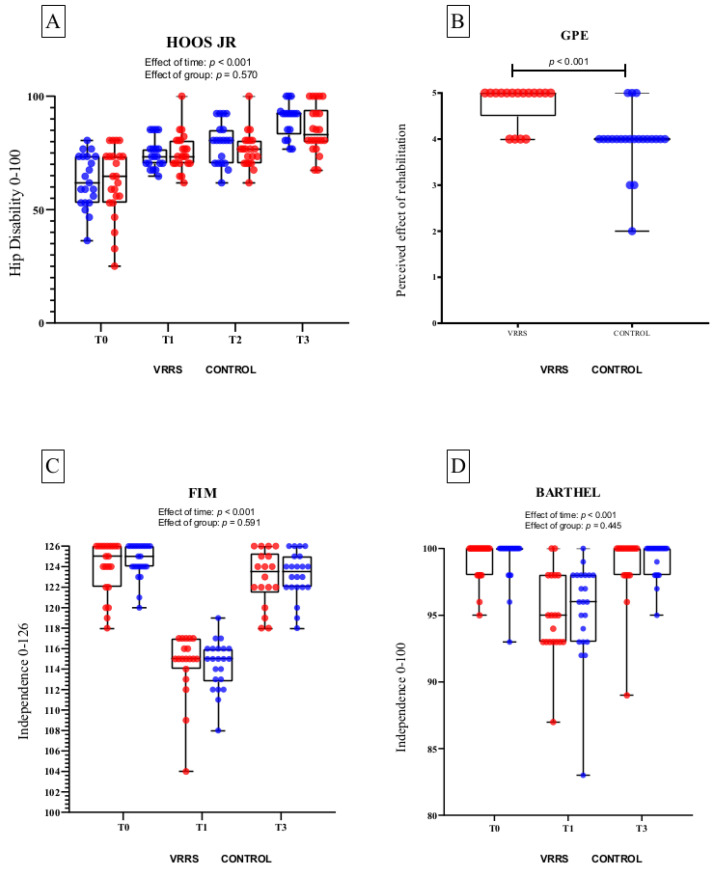
Whiskers box plots and single values of HOOS JR (panel **A**), GPE (panel **B**), FIM (panel **C**), and Barthel (panel **D**) scores for VRRS group (red circles) and control group (blue circles). HOOS JR: Hip dysfunction and Osteoarthritis Outcome Score for Joint Replacement (0–100); GPE: degree of perceived effect of the rehabilitation program (0–5); FIM: Functional Independence Measure (18–126); BARTHEL: Modified Barthel Index (0–100). Details on statistical differences for the effect of time are reported in Table 3.

The mixed-model ANOVA showed no significant group effect but a significant effect of time (*p* < 0.001) for all variables (Table 3). In detail, no difference was found in the HOOS JR between VRRS and control at T0 (*p* = 0.99), T1 (*p* = 0.99), T2 (*p* = 0.95), or T3 (*p* = 0.855). No differences in the secondary outcomes were found between the two groups at the different time points, with the only exception being for GPE at T3 (VRSS: 4.76 ± 0.43 vs. control: 3.96 ± 0.65; *p* < 0.001). Figure 4 shows the means and standard deviations for each variable at all time points. No differences were found between per-protocol and intention-to-treat analyses.

## 4. Discussion

Early rehabilitation after THA is essential for a proper functional recovery [23]; however, outpatient access to rehabilitation services following surgery may be limited by social, physical, or environmental barriers [24]. Telerehabilitation helps to overcome these problems by allowing treatments directly to the patient’s home. There are still very few studies investigating the effects of virtual-reality-based rehabilitation in orthopedics, and the results have highlighted that telerehabilitation following knee arthroplasty promotes levels of physical recovery comparable to conventional rehabilitation, along with high levels of patient satisfaction [25,26,27,28]. Nevertheless, to the best of our knowledge, this is the first RCT that has evaluated early virtual-reality-based home rehabilitation following THA.

The main result is that the virtual-reality-based home rehabilitation program resulted in similar improvements in functional outcomes with a better GPE compared to the traditional rehabilitation program within 15 days of THA. In detail, we did not detect significant differences between groups for the HOOS JR scale. This six-element, patient-reported outcome measure investigates pain and activities of daily living and provides a valid measure of hip health two years after THA, with a ceiling effect range of 36 to 46% [18]. This was evident from the mean scores of both groups on the 15th day after surgery. Therefore, the improvements in hip function after THA and rehabilitation were correctly detected by the HOOS JR, which did not give any further information about the impacts of the two different rehabilitation programs on hip functionality. Nevertheless, the effect of time indicated that both interventions (i.e., the virtual-reality-based and the traditional rehabilitation approaches) were able to improve hip functionality and reduce pain in patients undergoing THA for hip osteoarthritis. It is noteworthy that the telerehabilitation system, compared to the exercise booklet, allowed real-time monitoring of the patient’s rehabilitation activity and for motion data to be recorded. Evaluating the rehabilitation dose–response and dose–efficacy relationships is both a great opportunity and a challenge within the scope of telerehabilitation.

The FIM and Barthel scores showed high agreement in assessing and classifying the functional status of an individual based on the level of care they need [29]. Similarly, as previously discussed for the HOOS JR, a ceiling effect is noted for this pool of highly functioning subjects, who achieve total independence regardless of group allocation. An interesting finding was observed for GPE scores, with VRRS having a better-perceived effect for the rehabilitation program compared to control. In detail, participants perceived the exercise booklet as useful for the recovery after THA, while the experimental group considered the telerehabilitation system very helpful for their post-surgery functional recovery. Further, none of the participants felt uncomfortable with the VRSS device during the rehabilitation process. Finally, yet importantly, the human factor during the rehabilitation program, namely the operator who oversaw the post-surgical clinical course for VRSS daily and remotely, had a key role in subjects’ perceptions and satisfaction.

One of the major strengths of this study was the nature of its design (i.e., RCT), while the a priori sample size calculation allowed the correct statistical power to be reached for primary and secondary outcomes. Further, the study was conducted in a real-world environment, where the execution of virtual-reality-based rehabilitation and the application of new technologies in clinical practice represent huge challenges in the orthopedic field. The study represents a feasibility and effectiveness test of the application of a remotely guided home rehabilitation program through a telematic and user-friendly system. The use of data acquisition and recording systems, in compliance with severe privacy policies, can provide improved treatment strategies and can allow the detection of effective doses related to motor rehabilitation after surgery, such as THA [30,31]. Telerehabilitation may represent an advantage for the Italian National Health System by reducing the number of in-person sessions performed in hospitals or rehabilitation centers.

On the other hand, some limitations need to be acknowledged. First, the interventions for the two groups were unbalanced since the authors intended to compare clinical practice with optimal conditions. Consequently, we did not restrict what the control group did during the rehabilitation period. Second, we recognized and reported the redundancy and ceiling effect of the independence-level measurement scales, which are currently used in clinical practice. Third, it was impossible to conduct a double-blind trial due to the nature of the interventions; however, the subjects were asked not to reveal their treatment group to the outcome assessors, aiming to avoid detection bias. Unlike other studies in which telerehabilitation proved to reduce costs [6,32], we observed a possible opposite trend in our study. However, it is important to underline that it was beyond our study aims to evaluate the cost-effectiveness of the interventions.

## 5. Conclusions

In summary, a telerehabilitation system was used to oversee the home post-surgical course within 15 days of THA for patients with hip osteoarthritis. Similar to the standard of care, this virtual-reality-based system guided the patients in the experimental group to improve their hip health status as per the clinical goals for this type of intervention, as judged via patients having the highest scores on the perception of treatment efficacy rating scale. Nevertheless, although patients in the present study preferred the VRSS over unsupervised rehabilitation, the clinical outcomes were comparable for the two rehabilitation interventions. The telerehabilitation system has already been proven to be safe and effective, and in the middle of the COVID-19 pandemic, it has guaranteed remote accessibility to medical consultations, significantly reducing the risks associated with unnecessary travel and physical access to the hospital. Expanding some forms of healthcare practice beyond the physical boundaries of clinical health facilities is a cutting-edge strategy to respond to the growing demands for medical care. Cost-effectiveness studies on telerehabilitation need to be conducted in the future.

## Figures and Tables

**Table 1:** Home rehabilitation regimen after total hip arthroplasty. Control group I and VRRS group (V), respectively, received interventions through an illustrated booklet and the telerehabilitation system. “AMAYC“: “as much as you can”; 40 rep/5 min: 40 repetitions within 5 min; a.s.: after surgery; n/a: not applicable; VR: virtual reality technology.

	Exercise	Area	Mode	Group	Regimen	Tools
1	strengthening	Hip flexors	concentric	C	AMAYC	Illustrated exercise booklet
				V	40 rep/5 min (1st week a.s.), 80 rep/10 min (2nd week a.s.)	tablet and sensors, interactive VR exercise
2	strengthening	Hip extensors	concentric	C	AMAYC	Illustrated exercise booklet
				V	40 rep/5 min (1st week a.s.), 80 rep/10 min (2nd week a.s.)	tablet and sensors, interactive VR exercise
3	strengthening	Hip abductors	concentric	C	AMAYC	Illustrated exercise booklet
				V	40 rep/5 min (1st week a.s.), 80 rep/10 min (2nd week a.s.)	tablet and sensors, interactive VR exercise
4	strengthening	Hip adductors	concentric	n/a	n/a	n/a
				V	40 rep/5 min (1st week a.s.), 80 rep/10 min (2nd week a.s.)	tablet and sensors, interactive VR exercise
5	strengthening	Knee flexors	concentric	n/a	n/a	n/a
				V	40 rep/5 min (1st week a.s.), 80 rep/10 min (2nd week a.s.)	tablet and sensors, interactive VR exercise
6	strengthening	Knee flexors	auxotonic	C	AMAYC	Illustrated exercise booklet
				V	40 rep/5 min (1st week a.s.), 80 rep/10 min (2nd week a.s.)	tablet and sensors, interactive VR exercise
7	strengthening	Knee extensors	isometric	C	AMAYC	Illustrated exercise booklet
				V	40 rep/5 min (1st week a.s.), 80 rep/10 min (2nd week a.s.)	tablet and sensors, interactive VR exercise
8	strengthening	Knee extensors	concentric	C	AMAYC	Illustrated exercise booklet
				V	40 rep/5 min (1st week a.s.), 80 rep/10 min (2nd week a.s.)	tablet and sensors, interactive VR exercise
9	Load management	n/a	bipodalic	C	AMAYC	Illustrated exercise booklet
				n/a	n/a	n/a
10	Semi-quat	n/a	bipodalic	C	AMAYC	Illustrated exercise booklet
				V	40 rep/5 min (1st week a.s.), 80 rep/10 min (2nd week a.s.)	tablet and sensors, interactive VR exercise
11	Calf raise	n/a	bipodalic	C	AMAYC	Illustrated exercise booklet
				n/a	n/a	n/a
12	Lower Limb Triple Flexion	n/a	operated limb	C	AMAYC	Illustrated exercise booklet
				V	40 rep/5 min (1st week a.s.), 80 rep/10 min (2nd week a.s.)	tablet and sensors, interactive VR exercise

**Table 2:** Patient characteristics at baseline. Data are reported as means ± standard deviations. BARTHEL: Modified Barthel Index (0–100); FIM: Functional Independence Measure (18–126); HOOS JR: Hip Dysfunction and Osteoarthritis Outcome Score for Joint Replacement (0–100). * Chi-square test or *t*-test, as appropriate.

	Control Group (*n* = 23)	VRRS Group (*n* = 21)	*p*-Value *
Men—*n*. (%)	13 (56.5%)	12 (57.1%)	0.967
Age (years)	60.9 ± 7.52	61.5 ± 6.21	0.767
BARTHEL (0–100)	99.26 ± 1.71	99.19 ± 1.47	0.642
FIM (18–126)	124.57 ± 1.67	123.86 ± 2.63	0.612
HOOS JR (0–100)	62.23 ± 15.54	62.71 ± 11.74	0.909

**Table 3:** Between and within-group comparisons (VRRS and control groups) at different time points (T0, T1, T2, and T3), Data are reported as means ± standard deviations. HOOS JR: Hip Dysfunction and Osteoarthritis Outcome Score for Joint Replacement (0–100); FIM: Functional Independence Measure (18–126); BARTHEL: Modified Barthel Index (0–100). n/a: not applicable; ^a^: Tukey’s post hoc test, only non-significant comparisons are shown.

	VRRS Group				Control Group							
	T0	T1	T2	T3	T0	T1	T2	T3	Effect of Time	Contrast	Effect of Group	Interaction
HOOS JR	62.7 ± 11.7	74.6 ± 6.6	79.3 ± 9.3	89.7 ± 7.6	62.2 ± 15.5	75.5 ± 8.4	76.6 ± 7.9	85.4 ± 10.4	<0.0001	T1 vs. T2 (*p* > 0.05) ^a^	0.570	0.510
FIM	123.9 ± 2.6	114.4 ± 3.2	n/a	122.9 ± 2.7	124.6 ± 1.6	114.4 ± 2.4	n/a	123.2 ± 2.2	<0.0001	T0 vs. T3 (*p* > 0.05) ^a^	0.591	0.730
BARTHEL	99.2 ± 1.5	94.9 ± 3.1	n/a	98.6 ± 2.8	99.3 ± 1.7	95.4 ± 3.6	n/a	99.2 ± 1.3	<0.0001	T0 vs. T3 (*p* > 0.05) ^a^	0.445	0.835

## Data Availability

The study protocol was registered to ClinicalTrials.gov (NCT04221425).
